# Weekly team-based learning scores and participation are better predictors of successful course performance than case-based learning performance: role of assessment incentive structure

**DOI:** 10.1186/s12909-021-02948-6

**Published:** 2021-10-04

**Authors:** Gonzalo A. Carrasco, Kathryn C. Behling, Osvaldo Lopez

**Affiliations:** 1 Department of Biomedical Sciences, Cooper Medical School of Rowan University, 401 S. Broadway, Camden, NJ 08103 USA; 2 Department of Medical Sciences, Hackensack Meridian School of Medicine at Seton Hall University, 340 Kingsland Street, Nutley, NJ 07110 USA; 3 Present Address: Independent researcher, Houston, TX 77027 USA

**Keywords:** Academic achievement, Higher education, Medical education, Team-based learning, Microbiology, Infectious diseases

## Abstract

**Background:**

Incentives for preparation and participation in case-based (CBL) and team-based learning (TBL) differ by virtue of differences in assessment, allowing us to evaluate the role these incentives play in preparation and participation in these activities as well as overall course performance.

**Methods:**

Weekly TBL and CBL participation and performance as well as performance on the course final examination were recorded. Student participation was quantified and correlated with: (1) CBL preparation, participation, teamwork and completion of learning objectives scores, and (2) TBL individual readiness assurance test (iRAT) scores.

**Results:**

Student final examination scores (*n* = 95) were more strongly correlated with TBL than CBL performance. No significant correlation was found between iRAT and CBL scores. Student participation was measured in 3 CBL groups (8 students/group) and 4 TBL teams (6 students/team). TBL participation was more strongly correlated with final examination scores than CBL participation. TBL participation was also correlated with iRAT scores. CBL scores for preparation, participation, teamwork and completion of learning objectives did not significantly correlate with iRAT scores or TBL participation.

**Conclusion:**

These results suggest that the assessment incentives and methods used in TBL result in student performance that better predicts performance on summative examinations.

## Background

There are several benefits of active learning strategies described in the literature, including: improving student engagement and critical thinking, supporting a leaner-centered approach, assisting student identification of learning needs and resources, fostering peer teaching, and encouraging development of lifelong learning skills [1–5]. Importantly, two issues of extreme importance in these activities are optimal preparation and participation, where participation is often linked to the level of preparation of the learner [1, 6, 7].

**Fig. 1:**
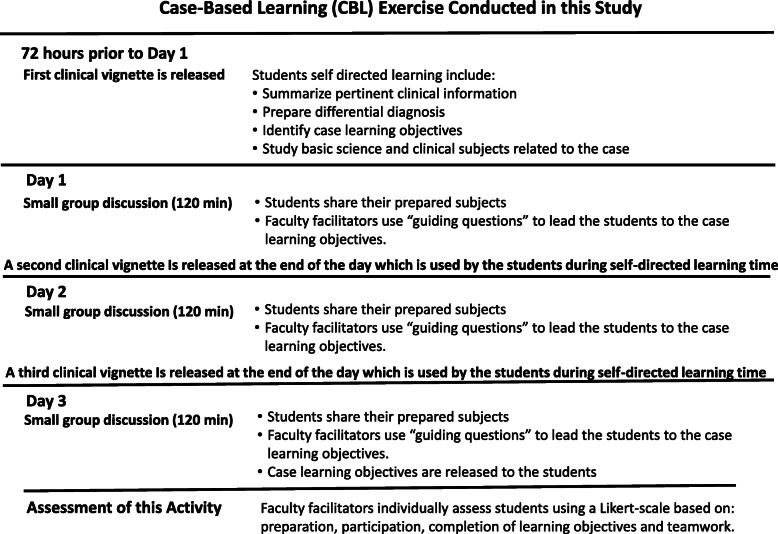
Simplified diagram of the Case-Based Learning (CBL) activity used in this study. This diagram identifies the activities conducted by the students prior to the small meeting. Students used clinical vignettes to prepare for each meeting. Importantly, Faculty facilitators individually assess students based on: preparation, participation, completion of learning objectives and teamwork

**Fig. 2:**
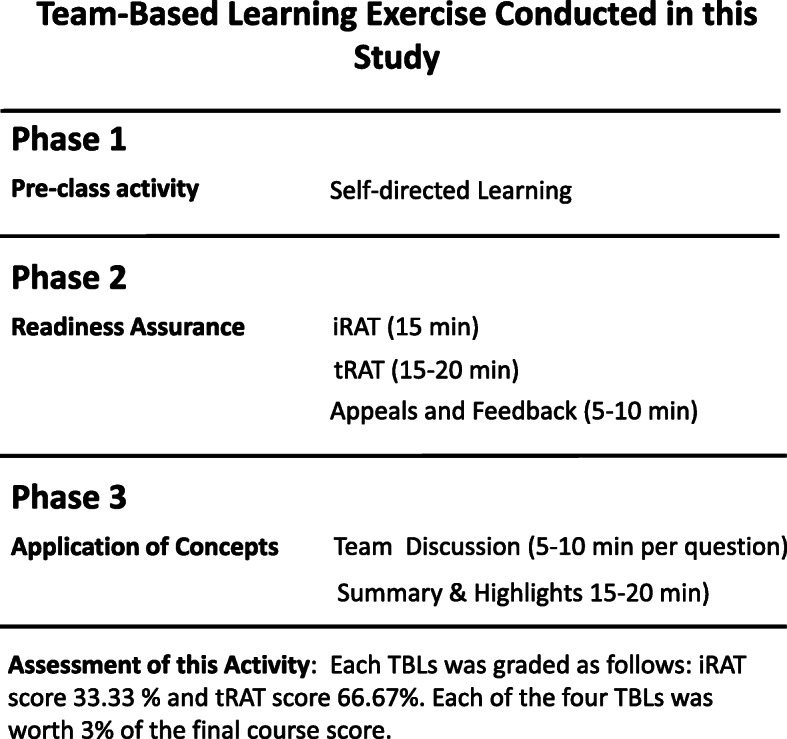
Simplified diagram of the Team-Based Learning (TBL) activity used in this study. TBL consisted of three phases. In Phase 1, students studied the content provided by the faculty facilitator prior to the activity. In Phase 2, students answered the individual- and team-readiness assessment tests (iRAT and tRAT respectively). A brief appeal and review process was conducted immediately after. In Phase 3, the last part of the activity, students discussed clinical case scenarios and/or experimental problems and were asked to apply the concepts they had just mastered during the tRAT. The activity ended after a facilitator-guided discussion to review the learning objectives included in this activity

At Cooper Medical School of Rowan University (CMSRU), in our integrated, pre-clinical curriculum, we use two active learning strategies with different incentive structures for participation: Active Learning Groups (ALG), a modified Case-Based Learning approach [8, 9], and Team-Based Learning (TBL) [10, 11]. The distinctive features of these learning strategies as they are utilized at CMSRU are explained in a recent review article [12]. Notably, in ALG, students use clinical vignettes and self-identified resources for preparation and are assessed for preparation, participation, completion of learning objectives and teamwork by two ALG facilitators (a basic science and a clinical faculty member) who may not be content experts for all the subjects that are discussed during the academic year [12] (Fig. 1). Conversely, in TBL, students prepare for the activity using learning material identified by the facilitator, which includes relevant didactic lectures, book chapters, laboratory material, etc. (Fig. 2). Students are assessed individually at the beginning of the activity through the individual readiness assessment test (iRAT), a 10-question, multiple choice quiz, and also during the team readiness assessment test (tRAT), that uses the same questions as the iRAT. Prior preparation and in-class participation influences tRAT scores as students discuss their answers before providing a team answer [12]. Subsequently, the students applied knowledge mastered during the tRAT to a series of more challenging multiple-choice questions, discussion of which was led by the TBL facilitator, during the application portion of the TBL exercise (Fig. 2). This was a not graded activity.

The goal of this study is to better understand how incentive structure affects student participation, and ultimately student performance, which is essential to proper implementation and possible improvement of these pedagogical techniques [13]. We have previously studied different strategies that would encourage students to improve their preparation and participation in TBL exercises. Indeed, we reported a significant reduction of course failures after implementation of graded TBL exercises at our institution [14]. We have also obtained improved performance and participation In TBL exercises by modifying the grading structure of the iRAT and tRAT exercises to create a new grading incentive [15]. These studies are in accordance with Haidet et al., who also investigated various strategies to motivate students for the tRAT portion of TBL exercises. Indeed they suggested that the incentive structure is one of the seven core elements of TBL implementation [6]. Furthermore, they stated that iRAT grading provides an incentive to enhance preparation prior to the TBL session whereas tRAT grading provides an incentive to maximize teamwork [6]. Accordingly, we have previously reported a positive correlation between student participation in TBL and overall course performance [16].

Our hypothesis for this study, is that differences in weekly incentives for participation and preparation in ALG and TBL lead to differences in individual student performance and participation as well as biomedical knowledge acquisition in an Infectious Diseases (ID) course for first year medical students.

## Methods

### Study setting

The ID course is a first-year medical school course, which reviews the application of microbiology and virology to human disease. There are four TBL exercises in this course. The first reviews basic science microbiology and immunology concepts discussed in the previous course, and the last three include topics taught during the first 3 weeks of the course. The final examination of medical knowledge, which takes place at the end of this course, is composed of multiple-choice questions in National Board of Medical Examiners (NBME) format requiring recall of facts and application of knowledge from the ID course.

**Fig. 3:**
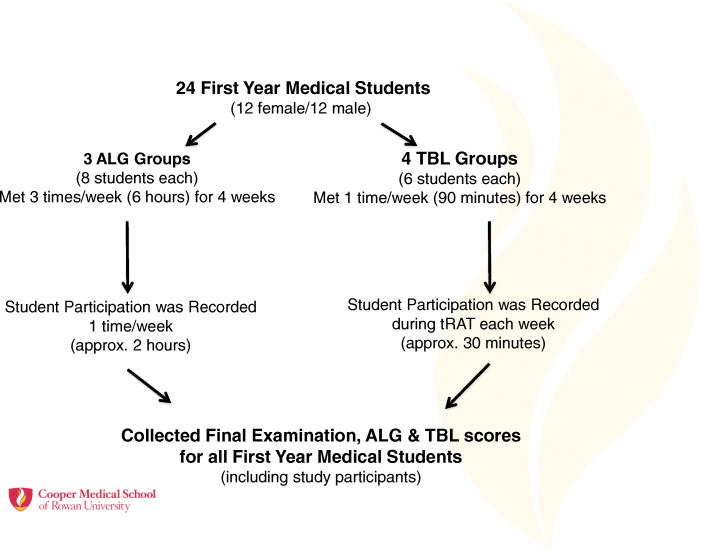
Schematic representation of the experimental design for the study. Twenty-four students from three different ALG groups were enrolled in this study. Two students from each ALG group were assigned to four TBL groups. Student participation was recorded once a week during ALG and TBL. Final examination, ALG and TBL scores for all the first-year medical students (*n* = 95), including the 24 enrolled participants were collected

Twenty-four first year medical students (12 female/12 male) were recruited through an informed consent process with prior IRB approval (PRO2016001096). The students were recruited from three different ALG groups (8 students per group). These groups met three-times per week during the 4 weeks of the ID course (Fig. 3). The same 24 students were assigned to four TBL groups (2 students from each ALG group) where they met once a week for the 4 weeks of the ID course. At the end of the course, ALG, TBL, and final examination scores were obtained for all the students (*n* = 95) including the 24 students enrolled in the study.

### TBL exercises

TBL exercises were executed as described above and following the guidelines described by Michaelsen and Sweet [10]. TBL team formation and student assessment were performed as previously reported [16], and included diversity in sex, undergraduate grade point average, undergraduate institution, race, and Medical College Admission Test (MCAT) scores. The student groups remained constant for the entirety of an academic year. TBL exercises were graded as follows: iRAT score 33.33% and tRAT score 66.67%. The final course score was calculated using the final examination score (88%) and the four TBL sessions (12% of total, 3% for each TBL).

### ALG exercises

ALG exercises were conducted as described above. ALG groups were composed of eight students and met three times a week for 2 h each session. ALG groups were assigned by the CMSRU Office of Students Affairs with the following considerations: Myers-Briggs personality testing, race, sex, national origin, prior academic achievement (MCAT scores and undergraduate GPA), and student undergraduate institution.

Students prepared for their ALG sessions by reviewing a clinical stem that was released 72 h prior to the first meeting of the week. In subsequent sessions, students received additional clinical information that they used to create a differential diagnosis, discuss ID-related basic science subjects, arrive at a final diagnosis for the case patient, and review target learning objectives at the end of the last session. As described above, ALG facilitators evaluated student performance weekly using a 5-point Likert scale (1 = unsatisfactory; 5 = excellent) in each of the following categories: preparation prior to ALG sessions, participation during ALG sessions, contribution toward group completion of case learning objectives, and ability to work as part of a team. Students were required to achieve an average score of at least 3 points to pass the ALG component of the ID course.

### Data collection and analysis

Final examination, ALG, and TBL scores for all students in the first-year class from the academic year of the study, including the 24 students participating in the study, were provided to the primary investigators by the CMSRU Office of Medical Education. The final examination consists of multiple-choice, factual questions requiring recall of information and application of knowledge learned in this course. An examination review committee, made up of faculty members with varied areas of expertise and the Office of Assessment, examines the psychometrics of each test question once a year to look for any problems. The Office of Assessment also compares student written examination performance to other national test performance standards evaluating correlations between internal and national assessments. This office also performs a thorough question item analysis to guarantee that examination questions fulfill internal reliability criteria over time. The reliability and validity of iRAT and tRAT questions, which have been used by several different cohorts of first year medical students, is manifested by the consistent performance of these question with different cohorts of students as we have previously published [16, 17].

Percentage of time participating in ALG and TBL for the 24 students enrolled in the study was collected with the help of paid study assistants who used an iPad application (Actio, SURRAtec, LLC) to record student participation during weekly ALG sessions and the tRAT portion of the weekly TBL exercises.

Mean averages and standard deviations for written final examination, ALG, and iRAT scores as well as percentage of participation were calculated and analyzed using IBM SPSS 26 (Armonk, NY). Changes in student scores were evaluated using Mann-Whitney statistical tests when appropriate. We also used a Pearson correlation coefficient (r) to assess the strength and direction of the linear association between two paired outcomes. *P* values are provided in the results. For all analyses, *p* values < 0.05 were considered statistically significant, and *p* values < 0.01 were considered highly statistically significant.

## Results

The average final examination, ALG and iRAT scores for the study group (*n* = 24) and the full class (*n* = 95) are shown in Table 1. Indeed, the final examination scores for the full class and study group were 85.40 (Standard deviation (SD): 6.68) and 85.84 (SD: 7.10) respectively, with no significant (*p* > 0.05) differences between the full class and study group. Similarly, no significant differences (*p* > 0.05) between the full class and study group were detected in either ALG scores (4.60 (SD: 0.40) vs. 4.42 (SD: 0.41), respectively) or iRAT scores (8.03 (SD: 1.59) vs. 8.23 (SD: 1.62), respectively).

**Table 1:** Final examination and active learning activity performance for the full class (*n* = 95) and study group (*n* = 24). No statistically significant (*p* < 0.05) differences were observed in these two experimental groups. Data is presented as average (standard deviation)

	Final Examination Score	ALG Score	iRAT Score
**Full Class (*n*** **= 95)**	85.40 (6.68)	4.60 (0.40)	8.03 (1.59)
**Study Group (*n*** **= 24)**	85.84 (7.10)	4.42 (0.41)	8.23 (1.62)
***P*value**	*P* > 0.05	*P* > 0.05	*P* > 0.05

Importantly, we detected a stronger correlation between final examination and iRAT scores for the full class (*r* = 0.379, *p* < 0.05) as compared to final examination and ALG scores (*r* = 0.178, *p* < 0.05) (Table 2). No significant (*p* > 0.05) correlation was detected between the full class ALG and iRAT scores.

**Table 2:** Fisher correlation coefficients (r) for the full class of first year medical students. Final examination scores are more strongly correlated with iRAT scores as compared to ALG scores for the full class (*n* = 95). Data is presented as average (standard deviation). *p < 0.05 and **p < 0.01

	r Full Class (*n* = 95)
**Final examination vs. ALG scores**	0.178*
**Final examination vs. iRAT scores**	0.379**
**ALG vs. iRAT scores**	0.139

The content discussed in ALG groups during 1 week of the course is tested in the TBL exercise on the first day of the subsequent week of the course. For instance, the ALG group discussion and course lectures during the third week of the ID course focused on Human Immunodeficiency Virus (HIV), antiretrovirals and immunocompromised patients, and the TBL that incorporated those subjects occurred on Monday of the fourth week of the course. As such, we investigated whether individual ALG scores during 1 week of the course correlated with iRAT performance in the subsequent week of the course for the full class of students (*n* = 95). A correlation between weekly individual ALG and final examination scores was also studied (Table 3). Weekly iRAT scores were highly significantly (*p* < 0.01) correlated with final examination scores in every week of the course (*r* = 0.48, *r* = 0.58, and *r* = 0.48 for the second, third and fourth weeks of the course, respectively) for the full class of students. We only detected a significant correlation (*r* = 0.22, *p* < 0.05) between final examination scores and ALG preparation scores during the second week of the course (Table 3).

**Table 3:** Fisher correlation coefficients (r) for ALG scores (preparation, participation, completion of learning objectives (LO), teamwork) or iRAT scores with final examination scores (full class, *n* = 95). While we detected a significant (*p* < 0.01) correlation between the second week of ALG preparation score and the final examination score, we found a consistent correlation (*p* < 0.01) between weekly iRAT scores and the final examination score. *p < 0.05 and **p < 0.01

	**Preparation** **Week 1**	**Participation** **Week 1**	**Completion of LO** **Week 1**	**Teamwork** **Week 1**	**Final Examination**
iRAT week 2	−0.09	−0.12	−0.17	− 0.02	0.48**
Final Examination	0.09	0.09	0.02	0.01	1
	**Preparation** **Week 2**	**Participation** **Week 2**	**Completion of LO** **Week 2**	**Teamwork** **Week 2**	**Final Examination**
iRAT week 3	0.04	0.09	−0.01	0.06	0.58**
Final Examination	0.22*	0.18	0.13	0.14	1
	**Preparation** **Week 3**	**Participation** **Week 3**	**Completion of LO** **Week 3**	**TeamworkWeek 3**	**Final Examination**
iRAT week 4	−0.09	−0.12	− 0.17	0.28	0.48**
Final Examination	0.09	0.09	0.02	0.01	1

We then investigated factors that could contribute to successful student academic performance in the different active learning activities and the course overall for the full class of students (*n* = 95). With this in mind, we compared final examination, ALG, and iRAT scores amongst students who achieved final examination scores in the upper (*n* = 31) and lower (*n* = 31) 33rd percentiles. As anticipated, final examination scores were highly significantly (*p* < 0.01) greater in the upper 33rd percentile as compared to the lower 33rd percentile (92.42 (SD: 2.16) vs. 77.55 (SD: 3.94) *p* < 0.01) (Table 4). While significant differences were detected between the upper and lower 33rd percentile in iRAT scores (8.70 (SD: 1.29) vs. 7.35 (SD: 1.68), respectively), no significant differences between these groups of students were detected when we compared average ALG scores (4.65 (SD: 0.40) vs. 4.54 (SD: 0.42) for the upper and lower 33rd percentile, respectively) (Table 4).

**Table 4:** Comparison of final examination, iRAT and ALG scores in students performing in the upper and lower 33rd percentile on the final examination (full class, *n* = 95). Students performing in the upper 33rd percentile on the final examination have higher final examination and iRAT scores but not higher ALG scores. Data is presented as average (standard deviation)

	Upper 33th Percentile (*n* = 31)	Lower 33th Percentile (*n* = 31)	*p*
**Final Examination**	92.42 (2.16)	77.55 (3.94)	< 0.01
**iRAT Score**	8.70 (1.29)	7.35 (1.68)	< 0.01
**ALG Score**	4.65 (0.40)	4.54 (0.42)	> 0.05

We then examined how participation of study students (*n* = 24) in either ALG or TBL related to final examination performance. We did this by recording the amount of time spoken during these activities as a surrogate for participation. We detected a highly significant greater student percentage of participation in TBL as compared to ALG (16.87% (SD: 8.87) vs. 12.50% (SD: 7.24), respectively). Importantly, when we evaluated the correlation between percentage of participation in TBL or ALG with final examination scores, we detected a stronger correlation between participation in TBL with final examination scores as compared to participation in ALG (*r* = 0.41, *p* < 0.01 vs. *r* = 0.24, *p* < 0.05, respectively).

We then compared percentage of participation in ALG with the subsequent week’s TBL percentage of participation and performance as explained above. The purpose of this analysis was to determine whether student participation in ALG during the week that is used to discuss the concepts tested in the subsequent week’s TBL influenced TBL performance and/or participation. Notably, the percentage of participation in TBL in weeks 2 and 4 significantly (*P* < 0.05) correlated with the iRAT scores during the corresponding week (*r* = 0.42 and *r* = 0.42 for TBL weeks 2 and 4, respectively). Our analysis also showed a stronger correlation between weekly percentage of participation in TBL and final examination scores as compared to ALG percentage of participation. Indeed, we found that the percentage of TBL participation in weeks 2, 3 and 4 significantly (*p* < 0.05) correlated with final examination scores (*r* = 0.41, *r* = 0.45, and *r* = 0.45 for TBL weeks 2, 3 and 4 respectively) (Table 5). Only during week 3 did we see a significant correlation (*r* = 0.47; *p* < 0.05) between percentage participation in ALG and final examination scores. No significant correlations between percentage of participation in ALG and ALG or iRAT scores was detected in this study.

**Table 5:** Fisher correlation coefficients (r) for ALG, iRAT and final examination scores with percentage (%) participation in either ALG or TBL (*n* = 24). We found a stronger correlation between weekly % participation in TBL, but not ALG, with iRAT and final examination scores in our study population (*n* = 24). *p < 0.05

	**% Participation in ALG** **Week 1**	**% Participation in TBL** **Week 2**
**ALG Score Week 1**	0.38	0.08
**iRAT Score Week 2**	0.03	0.42*
**Final Examination**	0.25	0.41*
	**% Participation in ALG** **Week 2**	**% Participation in TBL** **Week 3**
**ALG Score Week 2**	0.06	0.32
**iRAT Score Week 3**	−0.02	0.28
**Final Examination**	0.07	0.45*
	**% Participation in ALG** **Week 3**	**% Participation in TBL** **Week 4**
**ALG Score Week 3**	0.14	0.30
**iRAT Score Week 4**	0.28	0.42*
**Final Examination**	0.47*	0.45*

## Discussion

The hypothesis for this study was that differences in weekly incentives for participation and preparation in ALG and TBL lead to differences in individual student performance and participation as well as biomedical knowledge acquisition in an ID course for first year medical students. Our results support this hypothesis as we found that: (1) final examination scores were more strongly correlated with iRAT than ALG scores; (2) while weekly ALG scores did not correlate with iRAT scores from TBL sessions that incorporated the subjects discussed in those ALG sessions, weekly iRAT scores strongly and consistently correlated with final examination scores; (3) students performing in the upper 33rd percentile on the final examination had higher iRAT and final examination scores but no significant differences in ALG scores as compared to students performing in the lower 33rd percentile; (4) participation in TBL exhibited a stronger and more consistent correlation with iRAT performance, while no significant correlation was detected between participation in ALG and ALG performance; and (5) final examination scores significantly and consistently correlated with participation scores, while ALG participation lacked the same consistent correlation.

In active learning, incentive structure is a motivating factor, or reward, that drives the learning experience. Studies of incentive structure in medical education have been very limited and have mostly focused on a single active learning pedagogy in the medical education setting [18], which limits further interpretation and application of these findings in medical school curricula that incorporate multiple active learning strategies. Our studies at CMSRU, have focused on a comparative analysis between two commonly used and different active learning strategies, ALG and TBL, which have different incentive structures by virtue of different assessments for preparation and participation.

In ALG, a modified CBL setting, students are responsible for identifying their own learning resources; case learning objectives are provided by the case author at the conclusion of the case; and student assessment is completed by faculty facilitators that are not necessarily content experts for the weekly cases. In our experience, this incentive structure fosters teamwork and significant social competencies necessary for physicians after graduation, since the same group of students work through multiple, weekly, clinically-oriented cases throughout an entire academic year. Notably, ALG groups offer a non-judgmental venue where students engage in deep discussion of numerous biopsychosocial subjects during the academic year, which may not be discussed in other settings in our pre-clinical curriculum. Students also use this opportunity to share what they have learned in their own clinical experiences and to observe the behaviors of their preceptors, which they may want to emulate later in their careers. Finally, ALG-associated dialogue supports student development of diagnostic skills and empathy for patients while refining their professionalism skills. This will ultimately help them to provide better patient care by allowing them to connect with their patients and advocate on their behalf. However, as we have previously demonstrated, analysis of weekly ALG scores shows a lack of significant correlation between ALG assessments and improvement of cognitive skills, as measured in the course final examination [16]. These findings would demonstrate the importance of ALG for development of non-cognitive skills such as empathy and professionalism over acquisition of medical knowledge.

The incentive structure in TBL is quite different as previously described in the literature [11, 18]. TBL is classically divided into three phases, which include: 1) student preparation prior to the activity using learning materials identified by the TBL facilitator; 2) individual and team assessments used to encourage and demonstrate student mastery of the assigned learning materials; and 3) application of mastered concepts to course-related problems [11]. The data obtained in this study show a strong correlation between the weekly assessments of cognitive skills in TBL and final examination performance suggesting that the incentive structure in TBL provides tangible rewards for weekly pre-class preparation and therefore, academic success in both TBL and on the final course examination.

TBL also incentivizes teamwork and professionalism as students need to engage in relevant discussion of a course-related subject during the tRAT to arrive at a consensus answer. Therefore, the tRAT provides students with opportunities to develop both peer-teaching skills in a professional context and the ability to appropriately receive feedback from peers. Importantly, all the students in the group receive an immediate reward for their contributions to group discussion through use of the Immediate Feedback Assessment Technique (IF = AT) cards, which allow students to immediately see the correct answers for the iRAT/tRAT questions and consequently, which students have made the most valuable contributions to the discussion. This tRAT-associated incentive structure might be responsible for our current findings that indicate that weekly percentage of participation in TBL, but not ALG, strongly correlates with iRAT performance and more importantly with overall course performance as demonstrated by final examination scores. This may be because a significant driving factor for student participation in TBL is their knowledge of the subject under discussion, while in ALG there are other, as of yet, undetermined factors that are main contributors to participation. Therefore, changes in ALG incentive structure to provide more tangible rewards for preparation of course-related content prior to sessions may improve the educational impact of this method on acquisition of medical knowledge as we discussed elsewhere [12].

TBL and CBL techniques are frequently used in many medical schools around the world [3, 9, 19–21]. Therefore our studies have the potential to benefit and inspire medical educators around the world. The limitations of our analysis is that it was conducted in a single medical institution with a small number of enrolled students in the study group. Nevertheless, as noted above there were no statistical differences between the data collected for the small group and the full class, which would support the conclusions of this manuscript. Importantly, participation in TBL and CBL can be affected by the team dynamics. A survey conducted with the students prior and/or after the course could help our medical students to identify their skills and develop them based on feedback from their classmates. This kind of survey could also help us to better design the TBL teams in future studies to ensure that each team has a diverse mix of students (eg. background knowledge, gender, education, etc), as suggested by Burgess et al. [22]. Although this is an issue that we plan to address in future studies, representative student comments on the final ID course evaluation highlighted the benefit of teamwork in the TBLs conducted in this course: “I really liked having the TBLs each week. This held us accountable for the information and allowed us to work through material with classmates.”; and  "I love having TBLs, because I think any practice questions (and time to discuss with other students) is a benefit." Indeed, a working hypothesis for future studies could evaluate how students create their own incentives in these active learning activities which also might be higher in TBL, because there is a grade involved, than in CBL where there is less tangible consequence to poor preparation and participation.

## Conclusions

Implementation of active learning strategies has been backed by data that highlight significantly better student performance, deeper learning of course content, and improved critical thinking and application of medical knowledge compared to traditional pedagogical techniques [1, 23–25]. Our experience at CMSRU in the ID course, and more specifically with TBL, is consistent with these findings [26]. Additionally, TBL performance can be used for early identification of struggling students in need of additional support [17]. Nevertheless, different active learning pedagogies, used in parallel in a medical education setting, may provide a holistic approach to improve educational outcomes in undergraduate medical education [27] by preparing future physicians for life-long learning and excellent, empathetic patient care.

## Data Availability

The deidentified datasets used and/or analyzed during the current study are available from the corresponding author on reasonable request. The questionnaire used in final examinations and TBL exercises are part of our database and are not to be released.

## Abbreviations

TBL: Team-Based Learning
iRAT: Individual readiness assurance test
gRAT: Group readiness assurance test
ID course: Infectious diseases
ANOVA: Analysis of Variance
r: Pearson correlation coefficient
